# Evaluation of a web-based intervention to reduce antibiotic prescribing for LRTI in six European countries: quantitative process analysis of the GRACE/INTRO randomised controlled trial

**DOI:** 10.1186/1748-5908-8-134

**Published:** 2013-11-15

**Authors:** Lucy Yardley, Elaine Douglas, Sibyl Anthierens, Sarah Tonkin-Crine, Gilly O’Reilly, Beth Stuart, Adam W A Geraghty, Emily Arden-Close, Alike W van der Velden, Herman Goosens, Theo JM Verheij, Chris C Butler, Nick A Francis, Paul Little, on behalf of the GRACE consortium

**Affiliations:** 1 Centre for Applications of Health Psychology (CAHP), Faculty of Social and Human Sciences, University of Southampton, Highfield, Southampton SO17 1BG, UK; 2 Department of Epidemiology & Public Health, Health Behaviour Research Centre, UCL, Gower Street, London WC1E 6BT, UK; 3 Department of Primary and Interdisciplinary Care, University of Antwerp, Universiteitsplein 1 Wilrijk, Antwerp BE-2610, Belgium; 4 Primary Care and Population Sciences Division, Faculty of Medicine, University of Southampton, Aldermoor Health Centre, Southampton SO16 5ST, UK; 5 Julius Center for Health Sciences and Primary Care, University Medical Center Utrecht, Utrecht 3584 CX, The Netherlands; 6 Laboratory of Medical Microbiology, VAXINFECTIO, University of Antwerp, Antwerp, Belgium; 7 Institute of Primary Care and Public Health, School of Medicine, Cardiff University, Heath Park, Cardiff CF14 4YS, UK

**Keywords:** Prescribing, Antibiotics, Resistance, Questionnaires

## Abstract

**Background:**

To reduce the spread of antibiotic resistance, there is a pressing need for worldwide implementation of effective interventions to promote more prudent prescribing of antibiotics for acute LRTI. This study is a process analysis of the GRACE/INTRO trial of a multifactorial intervention that reduced antibiotic prescribing for acute LRTI in six European countries. The aim was to understand how the interventions were implemented and to examine effects of the interventions on general practitioners’ (GPs’) and patients’ attitudes.

**Methods:**

GPs were cluster randomised to one of three intervention groups or a control group. The intervention groups received web-based training in either use of the C-reactive protein (CRP) test, communication skills and use of a patient booklet, or training in both. GP attitudes were measured before and after the intervention using constructs from the Theory of Planned Behaviour and a Website Satisfaction Questionnaire. Effects of the interventions on patients were assessed by a post-intervention questionnaire assessing patient enablement, satisfaction with the consultation, and beliefs about the risks and need for antibiotics.

**Results:**

GPs in all countries and intervention groups had very positive perceptions of the intervention and the web-based training, and felt that taking part had helped them to reduce prescribing. All GPs perceived reducing prescribing as more important and less risky following the intervention, and GPs in the communication groups reported increased confidence to reduce prescribing. Patients in the communication groups who received the booklet reported the highest levels of enablement and satisfaction and had greater awareness that antibiotics could be unnecessary and harmful.

**Conclusions:**

Our findings suggest that the interventions should be broadly acceptable to both GPs and patients, as well as feasible to roll out more widely across Europe. There are also some indications that they could help to engender changes in GP and patient attitudes that will be helpful in the longer-term, such as increased awareness of the potential disadvantages of antibiotics and increased confidence to manage LRTI without them. Given the positive effects of the booklet on patient beliefs and attitudes, it seems logical to extend the use of the patient booklet to all patients.

## Introduction

The European Commission and the World Health Organisation have identified antibiotic resistance as a major public health concern. There is good evidence that antibiotic resistance is higher in patients prescribed antibiotics [1], that in EC countries where fewer antibiotics are prescribed there are lower levels of antibiotic resistance [2], and that reducing prescribing rates can reduce antibiotic resistance levels [3]. More than 80% of all antibiotics are prescribed in primary care, and at least 80% of these are probably unnecessary. In the case of acute lower respiratory tract infection (LRTI) – known by patients as a ‘chesty cough’ or ‘bronchitis’ – 80% of patients receive antibiotics, but the vast majority do not benefit significantly [4,5]: on average, patients receiving antibiotics will have a shorter illness duration by less than a day for a total illness duration of three to four weeks. There is consequently a compelling case for interventions to promote more prudent prescribing of antibiotics for acute LRTI. The study presented here is a process analysis of the recent GRACE/INTRO (Genomics to combat Resistance against Antibiotics in Community-acquired LRTI in Europe/INternet Training for Reducing antibiOtic use) trial of a multifactorial intervention to reduce antibiotic prescribing for acute LRTI in six European countries [6].

Research into GPs’ explanations for over-prescribing antibiotics has identified numerous contributory factors [7], including the view that antibiotics might help and will not harm the patient [8-10], and lack of awareness of the problem of resistance and the effects of their prescribing on resistance [11]. Deciding who will benefit from antibiotics is often difficult, and GPs will naturally prescribe as a precautionary measure when the risk of complications is seen as high [8,9,12]. Another common reason for prescribing is that the GP believes that the patient wants or expects antibiotics; this creates concern that failure to prescribe might leave the patient dissatisfied and damage the doctor-patient relationship, and that trying to persuade the patient that a prescription is not needed could take too much consultation time and might not be effective [9,10,12,13]. Although there is quite wide variation in prescribing rates between European countries [2], many of the factors influencing GP prescribing appear to be surprisingly similar across countries with very different prescribing contexts [14,15], suggesting that it might be possible to design interventions that could be implemented across Europe.

Simple, didactic educational interventions for GPs have not been shown to change prescribing behaviour, but effective approaches that the GRACE/INTRO trial drew on include multifactorial interventions, interactive educational methods and provision of patient education [16-19]. The GRACE/INTRO trial used a factorial design to compare two contrasting approaches, implemented in isolation and together. The first approach was training in use of the C-reactive protein (CRP) point of care test, which was intended to reduce diagnostic uncertainty and concern about risk of complications. The second approach was training in communication skills and use of a patient booklet, which was intended to help GPs feel more able to convince patients who did not need antibiotics that antibiotics were inadvisable. Both types of training were web-delivered and were preceded by an evidence-based rationale for reducing prescribing.

The GRACE/INTRO trial found that both intervention approaches were effective at reducing antibiotic prescribing, and the combination of both was most effective [6]. The purpose of the quantitative process study described here was to assess the views of doctors and patients of these interventions, to examine how they were implemented and what their effects were on GP and patient attitudes and satisfaction. An understanding of how the interventions were used and viewed is important for informing implementation in practice, while an analysis of the effects of the interventions on attitudes can provide insight into which ingredients may have been effective and in what ways. The Theory of Planned Behaviour [20] was used as the main framework for assessing GP attitudes as it has been shown previously to predict GPs’ intentions to prescribe antibiotics [8,21,22]. Research using the Theory of Planned Behaviour indicates that intentions and behaviour are influenced principally by the perceived positive and negative consequences of the behaviour and confidence in the ability to carry out the behaviour [23,24]. We also investigated whether there were country-specific differences in how the interventions were received, as these are also likely to affect the success of implementation across Europe [25,26].

## Methods

This was a quantitative process study nested within a cluster-randomised controlled trial. The study received ethical approval from ethics committees in all participating countries (i.e. Southampton and South West Hampshire Research Ethics Committee (A); Comité voor medische ethiek, Universitair Ziekenhuis Antwerpen; Medisch Ethische Toetsing Commissie; Clínic de Barcelona y secretaria del Comité Ėtico Investigación Clinica; Research and Ethics Committee of Primary Care Fundació Jordi Gol i Gurina; Komisja Bioetyki Universytetu Medycznego w Lodzi. All participants gave informed consent to take part in the study.

### Setting and participants

The trial was carried out in 229 practices in England, Wales, Belgium, the Netherlands, Spain and Poland. There were 424 GP usernames recorded on the web-based intervention (most GPs logged in individually, but some logged in as a group), and 346 GPs completed the self-report measures at either baseline, follow-up or both time points (the sample size therefore varies somewhat and is specified for each analysis). Of the 4,264 patients recruited to the study, 2,886 (67.7%) completed the self-report measures analysed here.

### Interventions

The web-based training was developed using the LifeGuide software [27], which allows development and easy modification (including translation) of web-based interventions without the need for programming. The web-based training consisted of a single session that drew on existing successful theory-based interventions to reduce antibiotic prescribing in primary care [17-19]. The session comprised three sections; an introduction, a module providing training in using a C-reactive protein point of care (CRP) test, and a module providing training in communication skills and use of a patient booklet. The communication skills training followed the STAR (Stemming the Tide of Antibiotic Resistance) model [17], aiming first to persuade GPs why a reduction in prescribing was necessary and then how it could be accomplished. The STAR model proposes that there are three elements of an effective consultation: to gather information, exchange information, and check information. Building on a recent successful website and booklet intervention to reduce prescribing for childhood respiratory infection [18], the GRACE/INTRO training also illustrated (using videos) how a patient booklet could be used in the consultation to address specific patient concerns; GPs were encouraged to use tick boxes in the booklet to highlight specific sections relevant to individual patients. The content of the patient booklet drew on previously validated content [28] that addressed perceptions of symptoms and antibiotics based on the extended Common Sense Model [29,30], which describes the dimensions of symptoms salient to patients (*i.e*., identity, cause, duration, severity of consequences, and potential for control or cure) as well as the salient dimensions of medication (*i.e*., perceived need and potential for harm). The materials were piloted in every country and modified according to feedback from interviews with health professionals and patients in each country, allowing small between-country differences in the website where this seemed advisable [31].

To encourage reflection on intervention implementation, during the training phase GPs were asked to document the presentation, management and issues raised by consultations for up to 10 cases in which the GP had attempted to implement the intervention. Where feasible, GPs were encouraged to participate in a one-hour seminar to share these experiences, and upload summaries of these discussions to an online forum.

The website was tailored to intervention group; all GPs in the intervention groups could access the introduction, but only GPs in practices randomised to the relevant groups could view the training module relevant to their intervention arm (*i.e*., CRP and/or communication skills). Further details of the intervention are given elsewhere [6]; the website can be viewed at https://www.lifeguideonline.org/player/play/intro_demo, and the patient booklet is provided as Additional file 1.

### Procedure

GP practices were cluster randomised to intervention group (for details of trial procedures, see trial paper [6]). GPs in the three intervention groups were sent a website address to login to the intervention, and were required to complete the baseline self-report measures when they logged on, before they could view the intervention. The post-intervention survey was administered to GPs in all groups by sending them an email invitation with a website address for completing the survey online (plus emailed reminders to non-respondents). Self-report measures were completed by patients at home as part of an illness diary, pro forma (returned by post), or a brief telephone-administered questionnaire.

### GP measures

Attitudes were assessed before and after the intervention by four items rated agree/disagree on a 7-point scale. Based on previous research into common reasons for prescribing [7,10-13], these items measured whether GPs thought reducing prescribing was a) important, b) risky, and c) could damage their relationship with patients, and how confident GPs were that they could reduce their prescribing. The post-intervention survey also asked whether GPs felt that taking part in the intervention had helped them to reduce their prescribing. The Website Satisfaction Questionnaire [28] comprised three items rated agree/disagree on a 10-point scale, that assessed whether GPs found the website helpful and trustworthy and the advice it provided sufficient. Self-report items post-intervention assessed whether GPs completed the online training individually or as a group and how they engaged in the reflective exercise. GP receipt of the intervention was measured objectively by the total duration spent viewing the intervention webpages.

### Patient measures

For simplicity, all patient self-report attitude measures were rated on a 10-point scale (strongly agree/strongly disagree). Patient perceptions of antibiotics were assessed by two items based on the two key dimensions of treatment perceptions in the extended Common Sense Model [29]: perceived necessity (‘It is usually necessary to take antibiotics to clear up a chesty cough’) and perceived harm (‘Taking antibiotics for a chesty cough can do more harm than good’). Patients were asked whether they had received a booklet and whether they found it useful. Patients then completed the Patient Enablement Instrument [32] and a three-item Consultation Satisfaction Questionnaire that assessed whether they felt they had received all the information and advice they needed and were generally satisfied with the consultation.

### Analysis

Since many self-report items were assessed by a single item, we did not replace missing data, but instead give the specific sample size for each analysis. All the scales employed had good internal reliability (Website Satisfaction Questionnaire, alpha = 0.93, n = 230; Patient Enablement Instrument, alpha = 0.92, n = 2,847; Consultation Satisfaction Questionnaire, alpha = 0.93, n = 2,888).

Effects of the intervention were analysed using General Linear Modelling (PASW statistics version 18) with univariate, multivariate or repeated measures as appropriate. Country differences were examined as post hoc exploratory analyses also using General Linear Modelling; the results could not be reported for every analysis but are given for key analyses and where significant variations between countries were found.

## Results

### GP intervention receipt and attitudes

The sample who completed the post-intervention survey comprised 147 men and 199 women with a mean age of 42.25 (s.d. 8.87), who had practised for a mean of 19.22 (s.d. 9.63) years. This sample represented 93.0% of the 372 GPs who supplied at least one case in the trial.

Most respondents reported completing the website training alone (189/230, 82.2%), but the remainder completed it as a group. The mean time members of the intervention groups spent on the website was 35.52 mins., with considerable variation (s.d. 28.12 mins.). Time spent on the website differed between groups [F (2,310) = 6.05, p =0.003], which was predictable as the communication skills training (26 core pages) was longer than the CRP training (15 core pages). The CRP group had the shortest duration (26.54 mins., s.d. 20.50), differing significantly on post hoc group comparisons from the communication group (37.44 mins., s.d. 28.94) and combined group (mean = 39.76 mins., s.d. 30.50). Of the 159 GPs who responded to the post-intervention question about how their seminar was organised, most took part in a practice-based seminar with multiple GPs (70/159; 44.0%), some engaged in self-reflection alone (28/159; 17.6%), a minority of practices met together (44/159; 27. 7%), and the remaining practices held a multi-practice teleconference.

**Figure 1:**
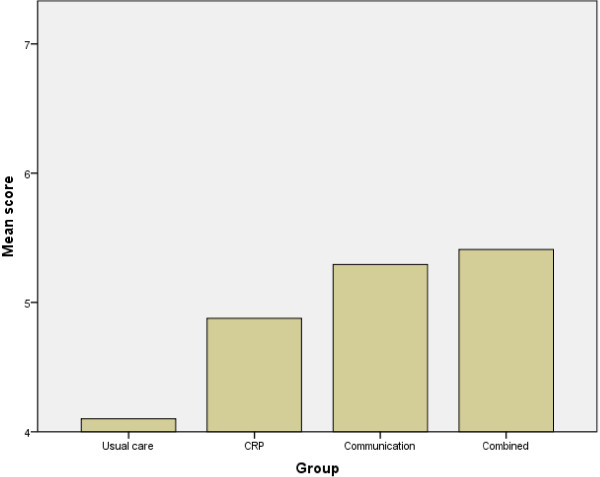
Mean scores on the item ‘Taking part in the study helped me reduce my antibiotic prescribing’ in the four intervention groups.

In the post-intervention survey there were clear group differences in overall perceptions of the extent to which taking part in the study had helped GPs reduce their antibiotic prescribing [F (3,287) = 11.06, p <0.001]; those in the communication and combined groups had the highest scores, and those in the control group the lowest (see Figure 1), with those in the CRP group intermediate. There were significant between-country differences in perceptions of study helpfulness [F (5,293) = 3.37, p = 0.006] and the helpfulness of the CRP test [F (5,150) = 6.89, p <0.001], though not in the perceived helpfulness of the booklet. Perceptions of the helpfulness of the study and especially the CRP test were highest in Spain and Poland (see Table 1). However, it is important to note that these findings are based on small numbers of respondents, particularly in Poland.

**Table 1:** Between country comparison of perceived helpfulness of the intervention

	**England**		**Wales**		**Belgium**		**Netherlands**		**Spain**		**Poland**	
	**n**	**Mean (s.d.)**	**n**	**Mean (s.d.)**	**n**	**Mean (s.d.)**	**n**	**Mean (s.d.)**	**n**	**Mean (s.d.)**	**n**	**Mean (s.d.)**
Taking part in the study has helped me reduce my prescribing	94	4.90* (1.50)	41	5.02 (1.64)	46	4.39 (1.58)	33	4.55 (1.25)	58	5.43 (1.33)	27	5.37 (1.82)
Using a point of care test has helped me reduce my prescribing	55	4.04 (1.75)	24	4.88 (1.48)	20	4.50 (1.57)	18	4.94 (1.63)	27	5.70 (1.41)	12	6.33 (4.88)
Using the GRACE/INTRO booklet has helped me reduce my prescribing	57	5.09 (1.43)	23	5.30 (1.40)	27	4.81 (1.44)	16	4.75 (1.29)	29	5.17 (1.58)	15	5.87 (1.41)

*Scores are on a scale from 1 (disagree strongly) to 7 (agree strongly).

Examination of changes in attitudes from baseline to post-intervention in the intervention groups (Table 2) confirmed that GPs saw reducing antibiotic prescribing as more important [F (1,226) = 15.23, p <0.001] and less risky [F (1,226) = 13.32, p <0.001] at follow-up (with no significant group differences). Reduction in perceptions of potential damage to relationships with patients did not quite reach significance [F (1,226) = 3.77, p = 0.054]. The main effect for increase in confidence to reduce prescribing was also not significant [F (1,226) = 2.55, p = 0.112], but there was a significant time by group interaction [F (2,226) = 3.21, p = 0.042]. Table 2 indicates that the interaction was due to a trend for those in the communication groups to gain confidence and those in the CRP group to lose confidence. Scores on the Website Satisfaction Questionnaire were generally high, with a mean of 8.26 (s.d. 1.52) out of a maximum score of 10, with no significant differences between groups or countries.

**Table 2:** GP attitudes to antibiotic prescribing at baseline and follow-up by intervention group

	**CRP group (n=73)**			**Communication group (n=73)**			**Combined group (n=83)**		
	**Baseline (mean (SD))**	**Follow-up (mean (SD))**	**Effect size (Cohen’s d)**^**a**^	**Baseline (mean (SD))**	**Follow-up (mean (SD))**	**Effect size (Cohen’s d)**	**Baseline (mean (SD))**	**Follow-up (mean (SD))**	**Effect size (Cohen’s d)**
Importance of reducing prescribing	6.03^b^ (1.27)	6.22 (1.00)	.15	5.85 (1.43)	6.34 (0.89)	.35**	5.90 (1.08)	6.25 (1.05)	.27*
Risks of reducing prescribing	4.37 (1.56)	4.88 (1.37)	.31*	4.81 (1.61)	5.16 (1.45)	.19	4.33 (1.47)	4.76 (1.50)	.23*
Risk to relationship with patients	4.49 (1.62)	4.63 (1.58)	.08	4.74 (1.80)	4.89 (1.61)	.08	4.76 (1.41)	5.12 (1.33)	.25*
Confidence to reduce prescribing	4.89 (1.49)	4.64 (1.49)	-.13	4.71 (1.79)	5.12 (1.70)	.21	4.86 (1.50)	5.28 (1.35)	.25*

*p < .05, **p < .01 for change in mean score (paired samples t-test).

^a^Effect size (d) reported such that positive value represents positive change.

^b^Scores are on a scale from 1 (disagree strongly) to 7 (agree strongly).

### Patient intervention receipt and attitudes

The patient sample comprised 1,024 (35.5%) men and 1,862 (64.5%) women with a mean age of 57.01 (s.d. 22.12). Of those in the communication and combined groups, 1,514/1,804 (83.9%) reported having been given a GRACE/INTRO booklet, most of whom also reported having used it (1,335/1,718; 77.7%). A third of those in the control and CRP groups (503/1,520; 33.1%) also reported having been given a booklet of some kind (presumably as a normal part of routine care), and a similar proportion (445/1,334; 33.4%) reported using it. Ratings of how useful the booklet was (where 7 indicates maximum usefulness) were relatively high in members of the communication groups who received the GRACE/INTRO booklet (mean = 5.69, s.d. 1.05), and were slightly higher [t (1,2034) = 6.99, p <0.001] than ratings for the booklets provided in the other groups (mean = 5.30, s.d. 1.18). According to the GP records, just over a third of patients in the CRP and combined groups received the CRP test (884/2,357; 37.5%).

Comparison of patient attitudes across countries (Table 3) revealed small but significant differences [F (4,2312) = 16.41, p <0.001]. On average, patients neither agreed nor disagreed that antibiotics for a chesty cough were usually necessary or that they could do more harm than good, with quite wide variability in attitudes. There were no significant country differences in antibiotics necessity beliefs, but beliefs about the potential harm of antibiotics were stronger among patients in Spain and Poland than in the Northern European countries [F (1,2315) = 11.11, p = 0.001]. Patients in Spain and Poland also reported greater enablement following the consultation [F (1,2315) = 22.87, p <0.001]. Satisfaction with the consultation was high in all countries, with no clear-cut pattern of geographical variation.

**Table 3:** Between country comparison of patient attitudes

	**England (n=276) mean (s.d.)**	**Wales (n=129) mean (s.d.)**	**Belgium (n=166) mean (s.d.)**	**Netherlands (n=201) mean (s.d.)**	**Spain (n=729) mean (s.d.)**	**Poland (n=856) mean (s.d.)**
Taking antibiotics is usually necessary	4.04* (1.61)	4.47 (1.73)	2.63 (1.76)	4.00 (1.57)	3.88 (1.76)	4.07 (1.72)
Taking antibiotics can do more harm than good	3.91 (1.35)	3.85 (1.46)	3.93 (1.32)	3.87 (1.31)	4.26 (1.55)	4.11 (1.49)
Patient enablement instrument	5.01 (1.02)	5.07 (0.93)	4.75 (1.19)	4.56 (1.10)	5.33 (0.92)	5.16 (1.03)
Satisfaction with consultation	5.93 (0.85)	5.99 (0.76)	6.07 (1.01)	5.56 (1.04)	5.96 (0.71)	5.80 (0.99)

*Scores are on a scale from 1 (disagree strongly) to 7 (agree strongly).

Comparison of patient attitudes across intervention arms (controlling for country effects) also revealed small but significant differences [F (12,6942) = 2.93, p <0.001]; mean scores are shown in Table 4. Beliefs that antibiotics were harmful did not differ between groups but beliefs that antibiotics were necessary were lowest in the CRP and combined groups [F (3,2315) = 5.43, p = 0.001]. Patients in the CRP group also reported slightly lower levels of enablement [F (3,2315) = 3.90, p = 0.009] and satisfaction with the consultation [F (3,2315) = 4.39, p = 0.004].

**Table 4:** Patient attitudes by intervention group

	**Usual care (n=433) mean (s.d.)**	**CRP group (n=584) mean (s.d.)**	**Communication group (n=659) mean (s.d.)**	**Combined group (n=644) mean (s.d.)**
Taking antibiotics is usually necessary	4.13* (1.74)	3.75 (1.71)	4.02 (1.80)	3.83 (1.72)
Taking antibiotics can do more harm than good	3.93 (1.43)	4.12 (1.46)	4.10 (1.51)	4.13 (1.45)
Patient enablement instrument	5.12 (1.03)	5.00 (1.06)	5.19 (0.95)	5.11 (1.08)
Satisfaction with consultation	5.85 (0.90)	5.76 (1.00)	5.95 (0.84)	5.89 (0.88)

*Unadjusted scores are shown, on a scale from 1 (disagree strongly) to 7 (agree strongly).

Specific effects of the interventions on patient attitudes were clarified by factorial analyses of group allocations and by examining the effects of actual receipt of the CRP test and booklet (again controlling for between country differences). Table 5 shows that allocation to one of the groups employing CRP testing resulted in lower antibiotics necessity beliefs, but actually receiving the CRP test did not. However, being allocated to one of the groups employing CRP testing resulted in slightly lower patient enablement scores, whereas actually receiving the CRP test resulted in lower enablement and lower satisfaction with the consultation. Conversely, being allocated to one of the booklet groups resulted in higher patient enablement and consultation satisfaction. Actually receiving the booklet resulted in lower antibiotics necessity beliefs and higher beliefs in the potential harm of antibiotics, as well as greater enablement and satisfaction with the consultation.

**Table 5:** Post hoc analyses of patient attitudes by receipt of CRP or booklet

	**Group allocation**			**Receipt of CRP test**			**Group allocation**			**Receipt of booklet**		
	**CRP group (n=1228)**	**Not CRP group (n=1092)**	**Effect size**^**a**^	**CRP test (n=884)**	**No CRP test (n=1473)**	**Effect size**	**Booklet group (n=1303)**	**Not (n=1017)**	**Effect size**	**Booklet (n=1453)**	**No booklet (n=856)**	**Effect size**
	**Mean (s.d.)**	**Mean (s.d.)**	**Cohen’s d**	**Mean (s.d.)**	**Mean (s.d.)**	**Cohen’s d**	**Mean (s.d.)**	**Mean (s.d.)**	**Cohen’s d**	**Mean (s.d.)**	**Mean (s.d.)**	**Cohen’s d**
Antibiotics necessary	3.79 (1.17)	4.07 (1.78)	.19***	3.90 (1.72)	3.94 (1.77)	.02	3.93 (1.76)	3.91 (1.73)	.01	3.87 (1.77)	3.99 (1.72)	.07*
Antibiotics harmful	4.12 (1.46)	4.03 (1.48)	.06	4.04 (1.45)	4.11 (1.47)	.05	4.12 (1.48)	4.04 (1.45)	.05	4.18 (1.48)	3.92 (1.45)	.18***
Patient enablement	5.06 (1.06)	5.16 (0.98)	-.10*	5.05 (1.05)	5.15 (1.02)	-.14**	5.15 (1.01)	5.05 (1.04)	.10**	5.17 (1.01)	5.00 (1.06)	.16**
Consultation satisfaction	5.84 (0.92)	5.91 (0.97)	.07	5.82 (0.94)	5.91 (0.87)	-.10*	5.93 (0.85)	5.81 (0.95)	.13***	5.92 (0.87)	5.79 (0.94)	.14***

*p < .05, **p < .01, ***p < .001 for between group differences (independent samples t-test).

^a^Effect size (d) reported such that positive value represents positive intervention effect.

## Discussion

Overall, GPs in all countries and intervention groups had very positive perceptions of the intervention and the web-based training, and felt that taking part had helped them to reduce prescribing. All GPs perceived reducing prescribing as more important and less risky following the intervention, and GPs in the communication groups reported increased confidence to reduce prescribing. Patients in the communication groups who received the booklet reported the highest levels of enablement and satisfaction and had greater awareness that antibiotics could be unnecessary and harmful.

It was interesting to find that the attitudes of both patients and GPs were most positive in the communication groups, particularly since the effect on antibiotic prescribing was actually greatest in the CRP groups [6]. A plausible reason could be that the CRP test was only relevant to and used for a minority of patients (*i.e*., cases of diagnostic uncertainty), whereas the communication skills training could be used with all patients [33]. However, patient enablement and satisfaction were actually lower in consultations where the CRP test was used, and there was a trend toward GPs losing confidence that the CRP test could help them reduce prescribing following experience of the intervention. Qualitative research suggests some possible explanations. GPs may have concerns about the reliability and interpretation of the CRP test in borderline cases, or experience practical difficulties with implementing the test, which could lengthen and disrupt the consultation [34-36]. Many GPs who have not used the CRP test believe that CRP testing could help them convince patients when antibiotics were not necessary [34,35], but the patients in this study viewed the CRP test as a useful tool for the GP that had no direct impact on them – whereas the patient booklet was highly valued for providing in-depth education about their symptoms and when they needed to consult (unpublished observations; paper in preparation). Previous qualitative research has also found that both GPs and patients are enthusiastic about the provision of written patient information explaining when patients are likely to receive meaningful benefit from antibiotics [37-39].

### Strengths and limitations

A strength of this study is that we used a theory-based approach and were able to document changes in beliefs and attitudes that have been shown to be relevant to GP and patient behaviour. A limitation of our design was that the control group did not access the website at baseline, and so their attitudes were not assessed pre-intervention. In addition, it was essential to minimise the burden on participating GPs and patients, and so only a few of the many potentially relevant constructs could be assessed, and we were obliged to use single items to measure some constructs (resulting in unknown measurement reliability for these measures). However, we also carried out qualitative process studies with both GPs and patients, and we note that the conclusions from our inductive qualitative research converge with the conclusions from the deductive quantitative research reported here (unpublished observations; papers in preparation).

It seems likely that there are complex multidirectional relationships between prescribing rates and the GP and patient attitudes we measured, and causal relationships could not be inferred from this study since attitudes were measured post-intervention. Although many of our findings were statistically significant, the effect sizes we observed were small. We were unable to analyse whether specific changes in GP attitudes mediated the effect of the intervention on prescribing rates because prescribing rates for individual GPs were not assessed prior to the intervention.

An innovative feature of the study is that it was carried out in six different countries, providing potentially useful information about the extent to which implementation of these interventions is likely to be influenced by local context. No negative views of the interventions were detected, but lower GP response rates in some countries (especially Poland) make it difficult to be sure that responses were representative of all countries. The practices that participated in this trial may not be typical and may have over-represented GPs who were particularly enthusiastic about reducing prescribing – although to minimise this risk, our recruitment criteria specified that only practices that had not previously taken part in a study of reducing antibiotic prescribing could participate, and prescribing in the baseline audit and the control group did not appear atypical.

### Implications for research and practice

Overall, these findings suggest that the interventions should be broadly acceptable to both GPs and patients across a range of European healthcare contexts and may be feasible to roll out more widely across Europe. The effectiveness of our interventions across quite different European countries might appear surprising given that different factors can influence prescribing in the different healthcare contexts; for example, a problem in some southern European countries is that patients can obtain antibiotics independently, while a problem in some Eastern European countries is the lack of clear prescribing guidelines [40]. However, there are also many shared influences on prescribing [14,15,40], and our process of co-design of the interventions by clinicians from every country, with minor country-specific modifications where necessary, may have been important. It is also possible that different elements of our complex interventions may have been effective in different countries or for different GPs; for example, the GP education element may have been more important for some and the patient booklet element more helpful for others.

There were some indications that the communication intervention might help to engender changes in GP and patient attitudes that will be helpful in the longer-term, such as increased awareness of the potential disadvantages of antibiotics and increased confidence to manage LRTI without them. There is a potential for experience of positive outcomes without antibiotics to reassure both doctors and patients that antibiotics are often unnecessary, which could reduce prescribing by doctors and also future consultation by patients [16,18,41]. However, there is also a possibility that patients could consult more often based on the safety-netting advice in the patient booklet [ref trial] or if they believe it is important to be given a CRP test to exclude serious infection [35,36]. A recent longitudinal follow-up of an intervention comparing the effects of communication skills training and use of the CRP test found that only communication skills training had longer-term effects on prescribing, while neither intervention significantly affected consultation rates [42]; however, further research is needed to confirm the longer-term effects of both interventions on prescribing and consultation rates.

Given that the CRP test was very effective in reducing antibiotic prescribing but had less positive effects than the booklet on patient beliefs and attitudes, it seems logical to extend the use of the patient booklet to patients receiving the CRP test, as well as those for whom the CRP test is not appropriate. The CRP test alone does not appear sufficient to maximise patient satisfaction and enablement, whereas receiving a booklet is likely to address patient concerns and provide effective reassurance [10,41]. In a previous study, patient satisfaction levels were higher in patients receiving the CRP test when those with intermediate test results were given a delayed prescription [36]. Future research could examine whether adding use of a delayed prescription to the CRP test and booklet (when appropriate) might optimise antibiotic use, patient satisfaction and re-consultation rates.

## Competing interests

The authors declare that they have no competing interests.

## Authors’ contributions

LY led the design of the study, consulting closely with HG, TJMV, CCB, NAF and PL, who made substantial contributions to the design of the survey. ED created the online survey and coordinated its translation and implementation, together with SA, ST-C, GO’R, AWV and AWAG. LY carried out the data analysis, with the assistance of BS, AWAG and EA-C. LY drafted the manuscript and all authors read and approved the final manuscript.

## Supplementary Material

Additional file 1 **Caring for coughs.** Your guide to managing chest infections. Click here for file
