# Supplementary material for: Evaluation of a web-based intervention to reduce antibiotic prescribing for LRTI in six European countries: quantitative process analysis of the GRACE/INTRO randomised controlled trial
Source: Implement Sci. 2013 Nov 15;8:134. doi: 10.1186/1748-5908-8-134 (PMC3922910; doi:10.1186/1748-5908-8-134)
Supplement: Additional file 1 — Caring for coughs. Your guide to managing chest infections. [file 1748-5908-8-134-S1.pdf]

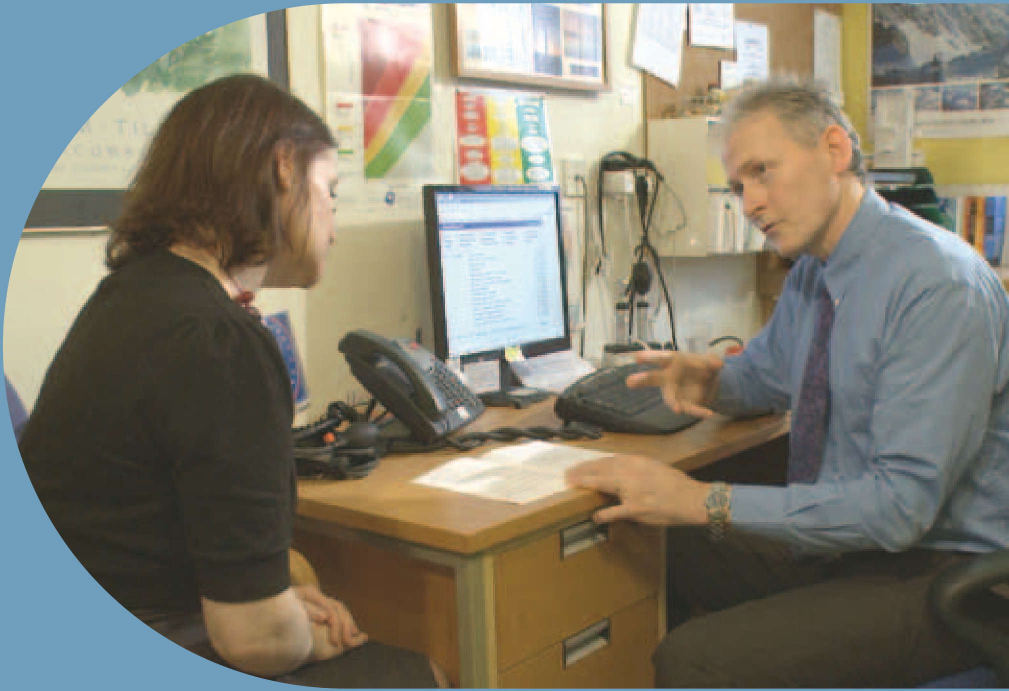

GRACE

Genomics to combat Resistance against Antibiotics in Community-acquired LRTI in Europe

Caring For Coughs:

Your guide to managing chest infections

How can this booklet help you?

Coughing is your body's way of clearing your airways, to keep them healthy. The problem is that coughs can last for a long time, and may be painful and disrupt your sleep, making you feel very unwell and interfering with your activities.

This booklet is designed to support explanation given during your consultation and we hope you will read it at home. It will give you the best up-to-date information you need about:

Page 3	What causes a cough?<br>When should my cough clear up?
Page 4	Would antibiotics help?
Page 6	What causes cough-related symptoms?
Page 7	Helping your immune system fight infection
Page 8	How you can care for your cough
Page 10	When should I seek further help?

☑ Throughout this booklet boxes appear next to each section. Your GP can use these to tick which sections he or she feels are most appropriate for you to read.

▢ What causes a cough?

You get a tickly cough when an infection in your throat makes it over-sensitive. The infection causes your airways (called 'bronchi') to get irritated, and often the nose and throat too. Coughing is the **normal way** for your body to try to remove whatever is blocking or irritating the airways. A tickly cough can be caused or made worse by viral infections (e.g. colds, flu), bacterial infections, hay-fever or other allergies, cigarette smoke, or air pollution.

Following a cold or flu virus it is also quite common for phlegm or mucus to develop, resulting in a 'chesty' cough. When the airways in your lungs become inflamed and irritated doctors will sometimes call this 'bronchitis'. Symptoms often include coughing up phlegm, discomfort or tightness in the chest, feeling tired, sore throat or runny nose.

A chesty cough (including bronchitis) lasting only a few weeks can be very unpleasant, but on its own it is not a dangerous or serious illness.

▢ When should my cough clear up?

Patients who see their doctor with a cough say that on average:

- their cough gets worse for up to 10 days after it starts
- it may remain bad with no sign of improvement for another week
- it then begins to slowly get better over the next 1–2 weeks.

A normal cough (including bronchitis) can easily take 3–4 weeks to clear completely and for some people (up to one in ten people) this can take up to 6 weeks.

▢ Would antibiotics help?

Antibiotics usually do not help people with acute cough, including bronchitis. People do not get better more quickly with antibiotics, even if the cough has lasted several weeks. Most common chest infections are caused by viruses, and research has shown that antibiotics do not work against most viruses. Most infections caused by bacteria also clear up at about the same rate whether treated with antibiotics or not.

Research has shown that on average antibiotics may help to reduce how long your cough lasts by only 1 day in an illness lasting 3–4 weeks.

Antibiotics can be helpful for some people with symptoms that won't go away and are getting worse. They are useful for some rare causes of coughs (e.g. pneumonia, and some types of infections caused by bacteria). Your doctor will prescribe antibiotics if you might have a more serious infection.

Taking antibiotics can be harmful

Using antibiotics too often causes bacteria to become 'resistant' to antibiotics. This means that bacteria that cannot be easily treated become more common. Some killer diseases are already resistant to several antibiotics, and soon we may not be able to find new kinds of antibiotics that still work.

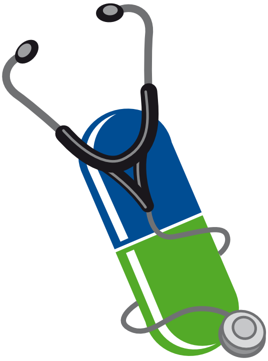

The main way to fight this problem is for doctors to use antibiotics only when they are really needed.

Taking antibiotics when you do not really need them puts you at unnecessary risk:

- If you have taken antibiotics you are more likely to have antibiotic resistant bacteria in your body, and to spread them to other people, such as your family. This means that you and your family are more likely to have an infection with a resistant bacteria the next time you are ill. When this happens antibiotics will work less well or not at all for you and your family.
- Taking antibiotics also reduces your natural defences against infection – by lessening your immune response, and by killing the natural 'good' bacteria in your throat that keep the harmful bacteria under control
- Antibiotics can cause side effects (such as diarrhoea, rashes, feeling sick), which in rare cases can be very severe (collapse, spasm of the airways).
- Always seek your doctors advice before taking antibiotics. Use of antibiotics without prescription can be harmful. They may be unnecessary, the wrong antibiotic for your current problem, out of date, or an insufficient course or dose.

So what can I do about my cough?

Your immune system will naturally clear up most common infections on its own. Scientific research has shown that there is nothing the doctor can do to speed up this recovery process. But your doctor – and this booklet – can give you advice on how to get relief from your symptoms and help your immune system to fight off the infection.

The next pages explain what may be causing your symptoms and what **you** can do to care for your cough.

▢ What causes cough-related symptoms?

Coughing up green phlegm or blood

Your body produces mucus or phlegm (green or yellow) as a normal reaction to inflammation in the airways to your lungs. The phlegm catches particles in your airways and helps keep your lungs clear.

Sometimes the mucus makes your breathing wheezy, or produces a rattling sound when you breathe or cough. Sometimes the phlegm in the airways is not loose enough to be coughed up easily – but your body will still manage to clear it out.

The phlegm may contain a tiny amount of blood. You should tell your doctor or nurse if you cough more blood than this or if you go on coughing up any blood, but most often it is quite normal.

Lying down can make a chesty cough worse because phlegm collects in the airways. But there is no risk of choking when sleeping as your body automatically makes you cough if you really need to – even if you have taken cough mixture to ease the cough.

Pain in the chest

Near the end of a cold, or after a sore throat or flu, it is quite common to feel discomfort or pain in your chest when breathing or coughing. Often this is because coughing has made the airways in your lungs inflamed.

Pain in the side of the chest is usually due to strained muscles – if this is the cause, the muscles will be painful when you press them. If you do not have tenderness in the muscles at the side of your chest, pain in the side of the chest can be due to the lining of the lungs being inflamed by a virus.

Severe pain in the chest, or having problems breathing as well as pain in the chest, can be a sign of more serious illness – see the section 'when should I seek further help?' at the end of this booklet.

Helping your immune system fight infection

This advice is based on evidence about how the immune system works.

- Drink enough fluids. The immune system needs normal fluids to work properly. The immune system is working hard to fight off the infection, so you need to make sure you are drinking enough – and when people are unwell they often eat and drink less without noticing. If you have any fever this increases your need for fluids as you will be sweating and may become dehydrated.
- Keep warm and get plenty of sleep.
- Eat a healthy, balanced diet containing plenty of fruit and vegetables. The body needs nutrients to fight off the infection.
- Stress can reduce the ability of the immune system to fight off infections. If you can reduce some of the stress in your daily life this will help to improve your mental and physical health.
- **Echinacea** is a natural herb that helps the body's immune system and studies show it helps the body fight infection and does help symptoms.
- There is some evidence that **Vitamin C** may help to fight viruses. The effects do not seem to be strong for everyone, but high doses of vitamin C may be helpful for people under physical stress (e.g. tired or cold).

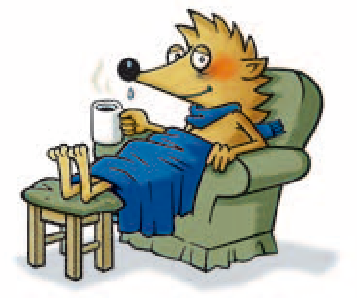

How you can care for your cough

Easing your symptoms without any medication

This advice is useful for all types of cough, but should work best for a tickly cough.

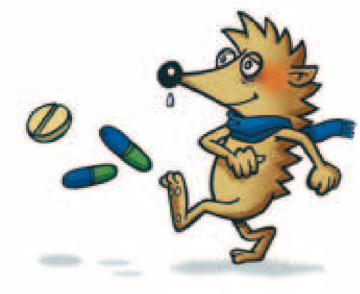

- You may find a simple and soothing cough syrup helpful. You can make one at home by mixing 2 parts honey with 1 part lemon juice – you can use it whenever you need to. Breathing dry air may make your cough worse.
- Try placing a bowl of water in front of the radiator to keep your room moist. Drink plenty of fluids to keep your throat moist. Taking hot showers or having a humidifier machine in the bedroom may also help.
- Avoid smoking or smoky or dusty atmospheres as these irritate the throat and chest even more.

Easing your symptoms with medication from the pharmacy

The aim of medicines you can get from the pharmacy is to try to ease the symptoms – these medicines do not help cure the infection and will not make your symptoms completely disappear.

There have been few studies to show whether or not taking cough medicines will ease the symptoms or shorten a cough, but some people do find they make their symptoms less troublesome. There are many types of medicines which have different effects, so we have suggested products which may help you.

Suppressing your cough

Sometimes the airways get over-sensitive even though there is not much to cough up. In this case calming or suppressing the cough may be helpful. You can suppress your cough by calming the brain's coughing centres – ask for cough mixtures containing a cough suppressant such as **dextromethorphan** (these products usually say they are for 'dry cough' or 'tickly cough'). Partly suppressing your cough will not harm the lungs nor delay recovery – you will still automatically cough up anything that really needs to be cleared out, even when you are asleep.

Making it easier to cough up phlegm

Cough medicines known as **expectorants** help to loosen the phlegm in your airways so you can cough it up more easily without pain or heavy coughing. A cough mixture that contains a **mucolytic** can also help to make the phlegm looser and easier to cough up. These products usually say they are for 'chesty cough' (and will often contain the ingredient **guafenesin**).

Easing pain

Pain in the chest is normally due to inflammation, so you can take **paracetamol**, and/or **ibuprofen***, which will help the pain and soothe the inflammation. You may also have muscular chest pain, sore throat, headache or fever – and all of these will be helped by paracetamol and/or ibuprofen.

** If you are asthmatic, have had stomach ulcers, or are on aspirin or steroid tablets it is best to avoid ibuprofen*

☑ When should I seek further help?

No guide can be complete. If you are worried about your symptoms after reading this leaflet then you should get advice. This could be telephone advice or a consultation with a doctor or nurse at your surgery. Telephone advice is also available from out-of-hours services (see contact numbers on the back of this leaflet). If you feel that it is an emergency you should dial 999 for an ambulance.

☑ Signs of possible serious illness – seek help at once:

- You are much more short of breath than normal – for example, you cannot talk in full sentences, you are unable to walk without getting short of breath, or your breathing is getting much worse (for example becoming more painful or breathing much faster than normal)
- High temperature (38.5°C or higher) that has lasted at least 4 days
- You are getting pain in the side of your chest when you breathe and muscles at the side of the chest are not tender
- Symptoms related to meningitis – unusually severe headache with any of these symptoms:
    - A stiff neck (difficulty putting chin to chest)
    - Discomfort or headache from bright lights
    - A rash (usually purplish spots) that does not fade with pressure (the rash will still be visible when the side of a clear glass is pressed firmly against the skin)
- Symptoms related to septicaemia (blood poisoning) – feeling <u>extremely ill</u> with any of these symptoms:
    - Uncontrollable shivering and/or drenching sweats
    - Severe pains in the arms or legs (for no obvious reason)
    - Feeling very weak, faint, sleepy or dizzy, so it is hard to stand
    - Unusual skin colour, looking pale, greyish or bluish, feeling cold and clammy to the touch

☑ Symptoms that should be assessed by your doctor – less urgent:

- A cough lasting more than 4 weeks without getting any better (or sooner if you are becoming more unwell or breathless much more easily)
- A fever of over 38. 5°C for 48 hours or more with no other sign of infection (cough, sore throat, runny nose, earache etc.)
- Unexpected weight loss along with a new cough or change in cough
- Coughing up blood which continues (a tiny amount of blood a few times is not unusual with a chest infection, but more than this means you should see your doctor)

☐ Seek help for your cough more quickly than other people:

- if you are over 65
- if you have asthma or diabetes
- if you have lung disease (e.g. chronic bronchitis, emphysema, chronic obstructive pulmonary disease COPD)
- if you have heart problems (e.g. heart attack, angina, heart failure)
- if you have a medical problem where your immune system is suppressed OR you are taking drugs that suppress the immune system (e.g. steroids, chemotherapy for cancer, some drugs used to suppress thyroid gland functioning)

Contacts:

GP's surgery phone number ....

GP or appropriate out of hours number ....

GP Stamp:

This booklet was developed by the GRACE network and is endorsed by:

EUROPEAN ANTIBIOTIC AWARENESS DAY
A European Health Initiative

CARDIFF UNIVERSITY
PRIFYSGOL CAERDYDD

UNIVERSITY OF Southampton

More information on GRACE can be found at www.grace-lrti.org
